# Phlebotomine sand flies (Diptera: Psychodidae) in the Greek Aegean Islands: ecological approaches

**DOI:** 10.1186/s13071-018-2680-4

**Published:** 2018-02-20

**Authors:** Nikolaos Tsirigotakis, Christoforos Pavlou, Vasiliki Christodoulou, Emmanouil Dokianakis, Christos Kourouniotis, Bulent Alten, Maria Antoniou

**Affiliations:** 1 Laboratory of Clinical Bacteriology, Parasitology, Zoonoses and Geographical Medicine, School of Medicine, University of Crete, Vassilika Vouton, GR-71003 Heraklion, Greece; 2 University of Crete, Vassilika Vouton, GR-71003 Heraklion, Greece; 3 Department of Biology, Faculty of Science, Ecology Division, VERG Laboratories, Hacettepe University, Beytepe-Ankara, Turkey

**Keywords:** Sand flies, Leishmaniasis, Fauna, Diversity, Abundance, Aegean islands, Greece

## Abstract

**Background:**

Blood-sucking phlebotomine sand flies are the vectors of the protozoan parasites *Leishmania* spp. Different *Phlebotomus* species transmit different *Leishmania* species causing leishmaniases which are neglected diseases emerging/reemerging in new regions. Thirteen sand fly species, ten belonging to the medically important genus *Phlebotomus* and three belonging to *Sergentomyia* are known in Greece. An increasing number of human and dog cases are reported each year from all parts of the country including the Aegean Islands. However, no previous study has been conducted on the sand fly fauna on the islands, except for Rhodes and Samos. The aim of this study was to investigate sand fly species in eleven small Aegean islands; to understand species-specific relationships with environmental and climatic factors and to compare sand fly community parameters among islands. A risk analysis was carried out for each species using climatic and environmental variables.

**Results:**

Nine sand fly species: *Phlebotomus neglectus*, *P. tobbi*, *P. similis*, *P. simici*, *P. perfiliewi*, *P. alexandri*, *P. papatasi*, *Sergentomyia minuta* and *S. dentata*, were collected from the islands studied. *Phlebotomus* (*Adlerius*) sp*.* and *Sergentomyia* sp. specimens were also collected but not identified to the species level. There was a positive effect of distance from the sea on the abundance of *P. neglectus*, *S. minuta* and *S. dentata*, and a negative effect on the abundance of *P. tobbi*, *P. simici* and *P. similis.* In general, temperature preferences of sand fly populations were between 21 and 29 °C. Nevertheless, there were significant differences in terms of temperature and relative humidity preference ranges among species. The most important species found, *P. neglectus*, was indisputably the most adapted species in the study area with a very high reaction norm, favoring even the lower temperature and humidity ranges*.* Overall, the sand fly fauna in the islands was very rich but there were differences in species diversity, as indicated by the values of the Shannon-Wiener index, along with evenness and richness of the sand fly fauna between the islands and altitude ranges in the islands.

**Conclusions:**

The study indicated that the Greek Aegean Islands, however small, maintain a rich sand fly fauna. This includes important vectors of *Leishmania* spp. representing a risk for parasite transmission to humans and dogs along with the danger of maintaining new *Leishmania* spp. if introduced to the area.

**Electronic supplementary material:**

The online version of this article (10.1186/s13071-018-2680-4) contains supplementary material, which is available to authorized users.

## Background

Phlebotomine sand flies (Diptera: Psychodidae, Phlebotominae) are medically important insects. The females are blood-feeding, able to sustain and transmit the parasitic protozoans *Leishmania* spp. (Kinetoplastida: Trypanosomatidae), the causative agents of leishmaniasis [1]; the bacterium *Bartonella bacilliformis* [2] and arthropod-borne viruses (phleboviruses and vesiculoviruses) [3–5]. The geographical distribution of sand fly species is the key factor determining the geographical distribution of these diseases. Although sand flies are active during the warm months, they spread their activity geographically and for longer periods of time during the year due to favorable climatic changes. This allows them, and the pathogens they carry, to reach new hosts and regions. Sand flies are small, delicate insects and use sheltered places to rest during the day, being active mostly at dusk and night [6]. They have a very strong adaptation capacity and a plastic reaction norm against changing environmental and climatic conditions. Even though they can fly relatively short distances from their breeding sites, some species have been reported to travel up to 2.3 km [7]. Sand flies are mostly found in rural and peri-urban environments and not at high altitudes since they require warm temperatures. They were, however, found in South America living in caves at 2800 m above sea level (masl) transmitting leishmaniasis [8, 9]. It is possible that an increase in genetic variation tracking the environmental (developmental temperature) change may have considerable implications on the distribution and range expansion of sand fly species, especially in warmer environments.

In Greece, 80% of the landscape is mountainous comprising an obstacle for the natural geographical spread of sand flies. At the same time, it has the twelfth longest coastline in the world (13,676 km long) and over 1400 islands, of which 227 are inhabited [9]. In Greece, thirteen sand fly species, ten belonging to the medically important genus of *Phlebotomus* and three belonging to *Sergentomyia* are known [10–15]; five of these are proven or suspected vectors of *L. infantum* (*P. perfiliewi*, *P. tobbi* and *P. neglectus*), *L. donovani* (*P. tobbi*), *L. tropica* (*P. similis*) and *L. major* (*P. papatasi*) in the Mediterranean basin.

Ecological studies show that the altitude and bioclimatic structure have an important impact on the distribution of sand fly species. Although altitude itself is not an ecological factor, it can act on sand fly distribution through the diversity of habitats, relief, and through the gradient of climate that it offers. The main objectives of this work were to investigate the sand fly fauna of eleven small islands scattered in the Aegean Sea between Greece and Turkey (acting as the transition area for most organisms between the two countries); to reveal sand fly relative abundance in positive traps; to compare the species found to those encountered in the two mainlands; to study the relationship of the species found to environmental and climatic factors; and to compare the islands in terms of some community parameters such as species diversity, dominance and species richness. A risk analysis was carried out for each species trapped in the islands using climatic and environmental variables. To the best of our knowledge, no previous study was conducted on the sand fly fauna in the Greek islands of the Aegean, except for Rhodes and Samos.

## Methods

### Study area, specimen collection and identification

**Fig. 1:**
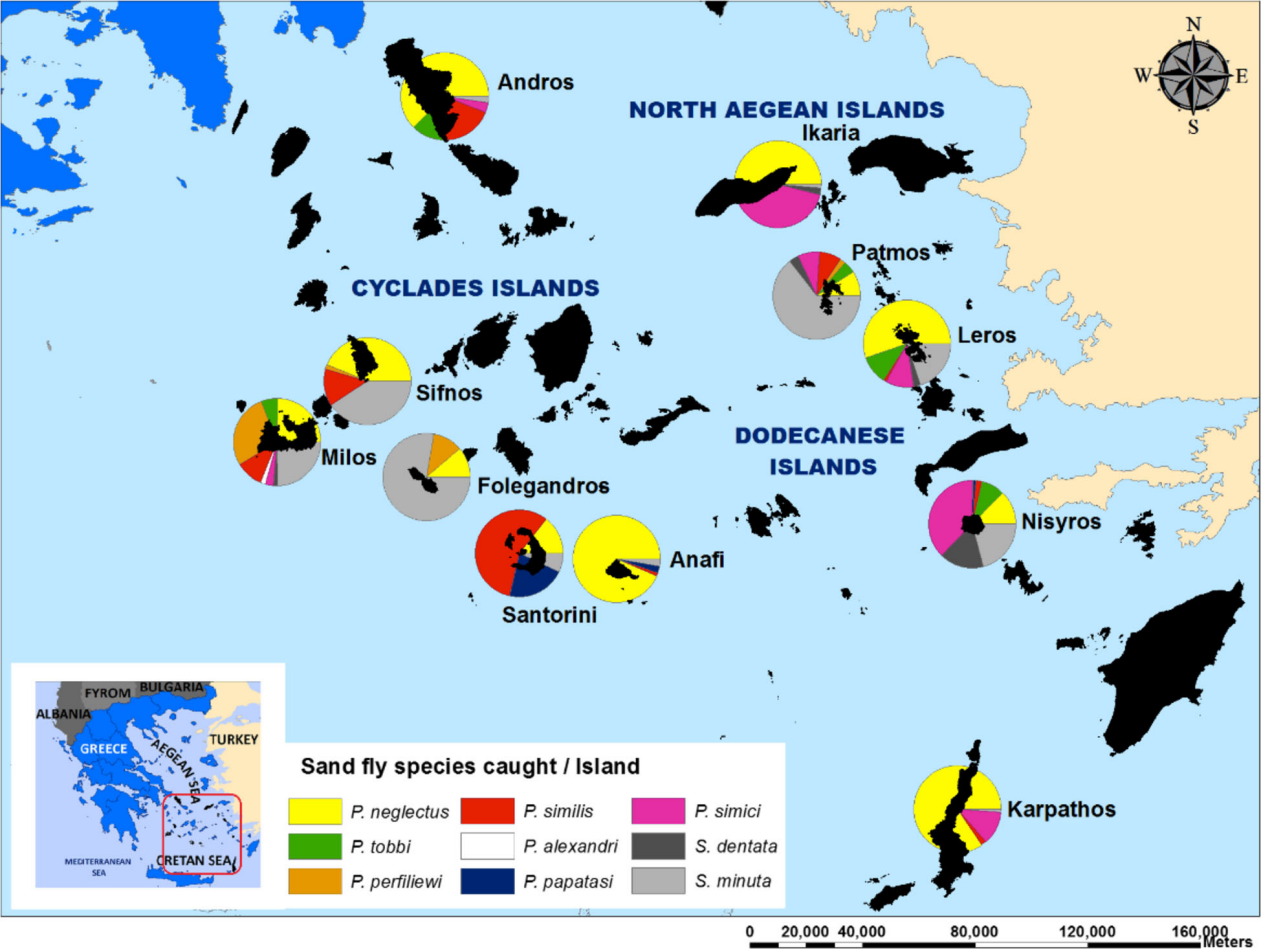
The occurrence of sand fly species in the eleven Aegean islands studied (the black outlines represent the islands)

Sand fly trapping was performed in eleven Greek Aegean Sea islands: Cyclades (Andros, Sifnos, Milos, Folegandros, Santorini, Anafi); Dodecanese (Patmos, Leros, Nisyros, Karpathos) and a North Aegean island (Ikaria). They are scattered in an area of 261 km by 258 km, forming stepping stones between Greece and Turkey (Fig. 1, Table 1).

**Table 1:** The geographical features of the eleven Greek Aegean islands studied

Island	Area (km^2^)	Latitude (°N)	Longitude (°E)	Max altitude (masl)	Human population^a^	Distance from Turkey (km)	Distance from Greek mainland (km)
Anafi	38.6	36.35841	25.82984	582	190	141.3	207.5
Andros	397.7	37.848228	24.908661	994	9170	126.0	11.4
Folegandros	32.4	36.65604	24.8507	415	780	209.5	132.2
Ikaria	255.0	37.61188	26.13851	1041	8423	57.0	135.6
Karpathos	302.2	35.63055	27.10905	1220	7310	94.5	355.0
Leros	54.1	37.15951	26.83281	320	7917	31.6	214.3
Milos	150.6	36.68548	24.50396	751	5129	244	105.5
Nisyros	41.3	36.59632	27.17521	698	987	17.5	275.3
Patmos	34.1	37.33087	26.54522	270	3047	49.0	187.0
Santorini	76.2	36.46056	25.39429	567	15,250	171.5	177.3
Sifnos	74.0	36.96325	24.72623	682	2442	220.2	88.0

^a^http://www.statistics.gr/en/2011-census-pop-hous

*Abbreviation*: *masl* metres above sea level

The climate in this area is Mediterranean (mild winters and dry, warm to hot summers with little rain) and the vegetation consists mainly of different shrub species. Overall, 265 traps were set in the eleven islands. Each trap was set for one night. Trapping was carried out for two to four nights per island according to island size and suitable habitats (Tables 1, 2). This allowed the comparison of sand fly species' presence and density from ecologically different areas. Sand fly sampling sites were chosen in relation to the presence of domestic animals (chicken, pigeons, ducks, geese, sheep, goats, horses, donkeys, rabbits, cats and dogs) on which sand flies may feed, or in unpopulated areas in order to trap sylvatic species as well.

**Table 2:** Sand fly trapping (using CDC light traps) performed in the eleven Greek Aegean islands studied

Island	No. of localities	No. of positive traps/total no. of traps	No. of trapping nights	No. of sampling sites with domestic/sylvatic animals	No. of sampling sites with rich/poor vegetation^a^	No. of indoor/outdoor traps	Date of trapping
Anafi	5	9/14	2	9/5	6/8	1/13	10-11/6/2016
Andros	12	12/20	3	9/11	4/16	1/19	11-13/6/2016
Folegandros	7	6/18	2	10/8	0/18	0/18	21-22/7/2016
Ikaria	13	15/26	3	10/16	7/19	0/26	15-17/6/2016
Karpathos	8	19/33	4	29/4	13/20	5/28	5-8/9/2016
Leros	9	18/18	3	12/6	4/14	3/15	21-23/6/2016
Milos	15	19/29	3	12/17	8/21	2/27	12-14/7/2016
Nisyros	6	28/28	4	1/27	20/8	2/27	24-27/6/2016
Patmos	10	16/21	3	12/9	4/17	3/18	18-20/6/2016
Santorini	8	5/28	2	22/6	1/27	7/21	13-14/9/2016
Sifnos	24	21/30	4	13/17	15/15	0/30	15-18/7/2016

^a^Rich vegetation implies the presence of trees and poor implies low levels or absence of vegetation

The biggest island studied was Andros (397.7 km^2^), and the smallest Folegandros (32.4 km^2^). Karpathos had the highest mountain, 1220 masl, followed by Ikaria at 1041 masl; all other islands had mountains lower than 1000 masl. The distance between islands varies from 20 to 287 km (Table 1).

Sand flies were collected during June, July and September 2016 using CDC miniature light traps (John W. Hock Co., Gainesville, FL, USA) which were operated between 06:00 pm and 06:00 am. A temperature/humidity data logger (LOG32 Temperature Humidity data logger, LOG32, Wertheim-Reicholzheim, Germany) was placed in close proximity to each trap, recording temperature (°C) and relative humidity (RH%) every thirty minutes for the time the trap was operating. Analyses were performed using the mean temperature and the mean relative humidity recorded during each night of trapping. A hand-held GPS (Magellan Triton 2000) was used to record the coordinates of each location. Measurements of wind speed were taken at the time the traps were set and collected using a Kestrel 1000 battery operated anemometer (Kestrel Instruments, Boothwyn, PA, USA). Data on the characteristics of each site were recorded in a questionnaire and photographs were taken of each trapping area. Captured specimens were collected next morning and kept in labeled plastic tubes, in 70% ethanol until processing.

The sand fly head, genitalia and wings were removed and mounted in CMCP-10 mounting medium (Polysciences, Inc., Warrington, PA, USA) while the rest of the body was stored in a newly labeled tube with 70% ethanol for molecular analysis. Species identification was carried out based on the morphology of the pharynx, male genitalia or female spermathecae and the wings [16, 17].

Five to six specimens of each species, in an equal sex ratio, were chosen and the DNA of each individual was extracted (Qiagen QIAamp DNA micro kit; Qiagen, Hilden, Germany) from the body parts remaining after the morphological identification. Subsequently, PCR reactions were performed targeting the barcoding region of the mitochondrial gene encoding cytochrome *c* oxidase subunit 1 (*cox1*) for animals using primers LCO1490/HCO2198 [18, 19] and/or the nuclear internal transcribed spacer 2 (ITS2) using primers JTS3/C1 [20]. The resulted PCR products were purified using Qiagen QIAquick PCR purification kit (Qiagen, Hilden, Germany). Double stranded sequencing was performed using the PCR primers by CeMIA SA (Larisa, Greece). Sequencing results (for the confirmation of the identified species) were checked by BLAST^TM^ queries. BLAST^TM^ queries indicated that, in all specimens, molecular and morphological identification agreed. In some cases (*Adlerius* and *Sergentomyia* specimens) identification to the species level was not possible due to the lack of sequence information, mostly when queries based on the barcoding (*cox*1) gene were performed. Therefore, these specimens were identified only to the subgenus level.

### Statistical analysis and mapping of the results

Analyses were performed, using SPSS 22 to determine the relation between the presence of sand fly species and ten environmental and climatic parameters such as: indoor/outdoor trapping; rural, sub-urban, urban areas; areas with or without domesticated animals; distance from the sea; altitude; vegetation type; presence of water source; average temperature at night of sampling; relative average humidity at night of sampling; and wind speed. Sand fly density was calculated as the percentage of each sand fly species per island/group of islands/all islands. Species with a low number of caught individuals were not considered in the analysis. Only significant statistical correlations by multivariate analysis are discussed. ArcGIS v10, GIS software was used for mapping the results.

Ecological comparisons between the islands and altitudinal ranges in terms of some community parameters (species diversity, evenness, dominance and richness) were performed using PAST, Palaentological Statistics, ver. 1.25 [21]. The Shannon-Wiener Species Diversity Index was used to calculate species diversity. The formulae and their rationale in the present study are summarized below.

Shannon-Wiener: H’=$$ \sum \limits_{i=1}^s(Pi)\left(\mathit{\log}2\right) Pi $$

where *S* is the number of species and *Pi* is the proportion of total samples belonging to *i*-th species.

Evenness: SHEI = H’/ln(S)

where H’ is the value of Shannon-Wiener Index, and S is the number of species in the sample (simple species diversity).

The distribution of sand flies, the environmental variables, the association between the presence/absence and the sand fly counts in positive traps were analyzed according to temperature and humidity ranges in each island studied. Negative binomial regression for over dispersed count data was employed to investigate the relationship between sand fly abundance in light traps and other explanatory variables including temperature, relative humidity, altitude and location (island). Interquartile ranges (25%, 75%) and the medians were calculated for positive traps at different altitude, temperature and humidity ranges.

## Results

### Sand fly fauna

**Fig. 2:**
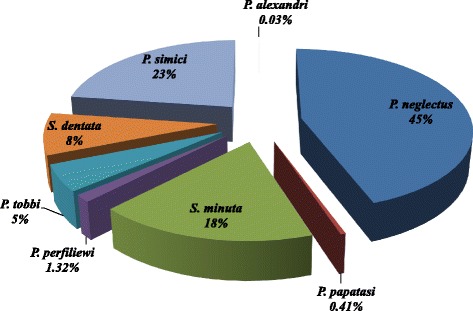
The overall percentage of total abundance for the nine sand fly species found in the eleven Aegean islands

Overall, 2957 specimens (1814 males and 1143 females) belonging to 9 sand fly species were collected on the 11 islands. Some *Phlebotomus* (*Adlerius*) and *Sergentomyia* specimens were also collected but they were not identified to species level due to a lack of DNA sequence information on the database. Therefore, only molecular results to the subgenus level assured the correct morphology in those specimens. *P. neglectus* was the most predominant species in the study area (1264 specimens; 42.7% of the total catch), followed by *P. simici* (642 specimens; 21.7 % of the total catch) (Table 3, Fig. 2). The volcanic island of Nisyros yielded the highest number of specimens (1219 specimens) and Folegandros the lowest (9 specimens). *Phlebotomus neglectus* and *S. minuta* were present in all 11 islands and *P. similis* in 10 out of the 11 islands (Table 3, Fig. 2).

**Table 3:** Number (*n*) and relative abundance (%) of collected sand fly species

Species	Anafi	Andros	Folegandros	Ikaria	Karpathos	Leros	Milos	Nisyros	Patmos	Santorini	Sifnos	Total		
	*n* (%)	*n* (%)	*n* (%)	*n* (%)	*n* (%)	*n* (%)	*n* (%)	*n* (%)	*n* (%)	*n* (%)	*n* (%)	*n* (%)	Male (%)	Female (%)
*Phlebotomus* (*Adlerius*) *simici*	0	4	0	82 (39.8)	70 (12.8)	19 (9.5)	4 (3.1)	453 (37.2)	9 (8.2)	0	1 (0.7)	642 (21.7)	45.5	54.5
*Phlebotomus* (*Larroussius*) *neglectus*	265 (94.3)	53 (61.6)	1 (11.1)	102 (49.5)	473 (86.2)	108 (54.3)	32 (24.4)	152 (12.5)	10 (9.1)	2 (11.8)	66 (44)	1264 (42.7)	73.0	27
*Phlebotomus* (*Larroussius*) *perfiliewi*	0	0	1 (11.1)	0	0	0	36 (27.5)	0	1 (0.9)	0	1 (0.7)	39 (1.3)	71.8	28.2
*Phlebotomus* (*Larroussius*) *tobbi*	0	11 (12.8)	0	8 (3.9)	0	21 (10.6)	8 (6.1)	103 (8.4)	5 (4.5)	0	0	156 (5.3)	62.2	37.8
*Paraphlebotomus alexandri*	0	0	0	0	0	0	1 (0.8)	0	0	0	0	1 (0.03)	100	0
*Paraphlebotomus similis*	1 (0.4)	16 (18.6)	0	6 (2.9)	5 (0.9)	2 (1)	13 (9.9)	27 (2.2)	9 (8.2)	8 (47.1)	21 (14)	108 (3.7)	45.4	54.6
*Paraphlebotomus papatasi*	7 (2.5)	0	0	0	0	0	0	2 (0.2)	0	3 (17.6)	0	12 (0.4)	75	25
*Sergentomyia minuta*	0	0	0	5 (2.4)	0	7 (3.5)	5 (3.8)	204 (16.7)	4 (3.6)	0	0	225 (7.6)	76.3	23.7
*Sergentomyia dentata*	8 (2.8)	2 (2.3)	7 (77.8)	3 (1.5)	1 (0.2)	42 (21.1)	32 (24.4)	278 (22.8)	72 (65)	4 (23.5)	61 (41)	510 (17.2)	54.9	45.2
Total	281	86	9	206	549	199	131	1219	110	17	150	2957	61.4	38.65

The altitudinal distribution and abundance data for all species collected from the study area are shown in Table 4. Whilst the majority (93.4%) of all specimens were collected at an altitude range of 0–400 m, 61.6% of the sand fly populations were collected between 0–200 m and 77.4% between 0–300 m. The most important vector of human visceral leishmaniasis (VL) and canine leishmaniasis (CanLei), *P. neglectus*, was collected at all altitudinal ranges below 600 masl, and its distribution showed a very strong negative correlation with altitude (*r* = -0.78, *P* = 0.01). The highest densities of this species were registered at altitudes of 0–100 masl (total catch: 506). In contrast, among all species, the distribution of *S. dentata* showed a strong positive correlation with altitude (*r* = 0.76, *P* = 0.05) with the highest number of specimens at the 101–200 masl range. *Phlebotomus tobbi* was also found at all altitudinal ranges below 600 masl. Its distribution showed a weak negative correlation with altitude (*r* = -0.28, *P* = 0.05). No sand fly specimen was collected above 600 masl in the study area.

**Table 4:** Number (*n*) and relative abundance (%) of the sand fly species collected in relation to altitude

Species	Altitudinal range (masl)														
	0–100		101–200		201–300		301–400		401–500		501–600		> 600		Total
	*n*	(%)	*n*	(%)	*n*	(%)	*n*	(%)	*n*	(%)	*n*	(%)	*n*	(%)	*n*
*P. simici*	142	22.1	140	21.8	104	16.2	211	32.9	14	2.2	31	4.8	0	0	642
*P. neglectus*	506	40.0	371	29.4	158	12.5	170	13.4	6	0.5	53	4.2	0	0	1264
*P. perfiliewi*	7	17.9	31	79.5	1	2.6	0	0	0	0	0	0	0	0	39
*P. tobbi*	49	31.4	46	29.5	30	19.2	26	16.7	3	1.9	2	1.3	0	0	156
*P. alexandri*	1	100	0	0	0	0	0	0	0	0	0	0	0	0	1
*P. similis*	26	24.1	24	22.2	42	38.9	14	13.0	0	0	2	1.9	0	0	108
*P. papatasi*	6	50.0	6	50.0	0	0	0	0	0	0	0	0	0	0	12
*S. dentata*	26	11.6	185	82.2	11	4.9	2	0.9	1	0.4	0	0	0	0	225
*S. minuta*	152	29.8	106	20.8	120	23.5	51	10.0	11	2.2	70	5.9	0	0	510
Total	915	30.9	909	31.0	466	16.0	474	16.0	35	1.2	158	5.3	0	0	2957

*Notes*: 61.6% sand flies were captured between 0-200 masl; 77.4% between 0–300 masl and 93.4% between 0–400 masl.

### Comparison of the islands in terms of sand fly communities

Species composition differed between islands which may reflect the changes in the environment of the wider area. Overall, *P. neglectus* (proven vector of *L. infantum*) and *S. minuta* were collected from all islands (Table 3). One member of the subgenus *Larroussius*, *P. tobbi*, the proven vector of *L. infantum* and also of *L. donovani* in Turkey and Cyprus, was found to be predominant in the islands closer to Turkey in the East, while *P. perfiliewi* (proven vector of *L. infantum* in the Mediterranean) was present mostly along the islands on the western part of the Aegean Sea (closer to Greece). Patmos, Nisyros, Leros (on the East) and Milos (on the West) may be considered transitional islands since they are more influenced by the mainlands, Greece and Turkey (Fig. 1, Tables 3, 5). Even though there were differences between the islands, with the range of 3–8, the species richness was found to be very high in most of the islands. There were also differences in species diversity, as indicated by the values of the Shannon-Wiener index (H’), and evenness among the islands (Table 5). Because of their relatively high microhabitat diversity, all three parameters were found to be higher in four islands (Patmos, Nisyros, Leros and Milos), whilst species diversity (1.66), evenness (0.80), and richness (8) were found to be maximal in Milos, the island with the lowest dominance index (0.213). Due to *P. neglectus* dominance, the lowest diversity (0.27) and evenness (0.173) was determined in Anafi with the highest dominance index (0.89).

**Table 5:** Biodiversity analysis for the eleven Greek Aegean islands and different altitude ranges

Island	Richness (*R*) ^a^	Diversity (Shannon Entropy)		Evenness (*H*/ln*S*)	Simpson dominance	
		*H* (nat)	*H* (bit)		SD	SD (unbiased-finite samples)
Anafi	4	0.278	0.402	0.173	0.891	0.890
Andros	5	1.079	1.557	0.671	0.443	0.436
Folegandros	3	0.683	0.986	0.622	0.630	0.583
Ikaria	6	1.095	1.508	0.612	0.407	0.404
Karpathos	4	0.445	0.642	0.321	0.759	0.758
Leros	6	1.252	1.806	0.699	0.371	0.368
Milos	8	1.666	2.404	0.802	0.223	0.213
Nisyros	7	1.557	2.246	0.869	0.245	0.244
Patmos	7	1.208	1.743	0.621	0.454	0.449
Santorini	4	1.116	1.610	0.805	0.398	0.352
Sifnos	4	1.139	1.643	0.708	0.371	0.367
Total evaluation	9	1.533	2.212	0.698	0.276	0.276
Altitude (masl)						
0–100	9	1.311	1.891	0.597	0.364	0.363
101–200	8	1.628	2.349	0.783	0.239	0.239
201–300	8	1.439	2.076	0.692	0.290	0.288
301–400	6	1.356	1.957	0.757	0.304	0.302
401–500	5	1.109	1.389	0.814	0.315	0.294
501–600	4	0.725	1.045	0.523	0.570	0.567
601–700	0	0	0	0	0	0

^a^Only identified species were summed

Between the altitudes of 100 and 200 masl, species diversity was more significant (Table 5). The richness, diversity, and evenness were maximal between 100–200 m (S = 8; H’ = 1.628; E = 0.783) and 200–300 masl (S = 8; H’ = 1.439; E = 0.692). Although the highest richness was found at 0–200 masl altitude (S = 9), diversity (H’ = 1.311) and especially evenness (E = 0.597) was relatively low. Diversity and species richness were found to be lower at the altitudinal ranges of 400–500 masl (H’ = 1.628; S = 5) and 500–600 m (H’ = 0.725; S = 4) being the most critical elevations for dispersing local populations. No sand fly specimen was collected above altitude of 600 masl. Evaluation of the results from community analysis suggested two altitudinal assemblages directly associated with sand fly fauna, one at relatively higher altitudes between 500–600 masl (negative effect) and one at lower altitudes of 0–300 masl. The transition zone between these two assemblages is in the range of 400 mals.

### Risk factors for the presence of sand flies in the Greek islands

Ten risk factors were associated with the presence of phlebotomine sand fly species by bivariate regression models followed by multivariate analysis. Only the results of the multivariate analysis are discussed (Additional file 1: Table S1). *Phlebotomus neglectus* had a significant positive correlation related to trapping sites found less than 1000 m distance from the seashore (*P* < 0.0001), and although it was found at 0–500 m altitude it was favored by altitudes lower than 200 m (*P* = 0.008), rural areas (*P* < 0.0001), temperatures below 30 °C and closer to 21 °C (*P* = 0.007), and relative humidity over 60% (*P* < 0.0001). *Phlebotomus tobbi* showed preference for relatively lower temperatures between 24 and 27 °C (*P* = 0.001) and was found mostly outdoors (*P* = 0.022). This species was collected in very low numbers at altitudes higher than 300 masl (Additional file 1: Table S1). *Phlebotomus simici*, on the other hand, was favored by altitudes above 400 masl (*P* < 0.0001), outdoors (*P* < 0.0001), a relative humidity of 30–60% (*P* < 0.0001) and temperatures of 29–30 °C (*P* < 0.001) and was found at even higher temperatures than 30 °C. *Phlebotomus similis* showed positive correlation with urban areas (*P* < 0.0001) and relative humidity of 30–60% (*P* = 0.001); this species was found mostly indoors (*P* < 0.0001) and at relatively lower temperatures (*P* = 0.006).

*Sergentomyia minuta* was positively correlated to a distance from the seashore < 1000 m (*P* < 0.0001), urban areas (*P* < 0.0001), indoor (*P* < 0.0001), temperatures around 29–30 °C (*P* = 0.003) and a relative humidity of 30–60% (*P* < 0.0001). *Sergentomyia dentata* was positively correlated with a distance from the sea higher than 2000 m, altitudes around and lower than 200 m, indoors and relative humidity lower than 60%.

**Fig. 3:**
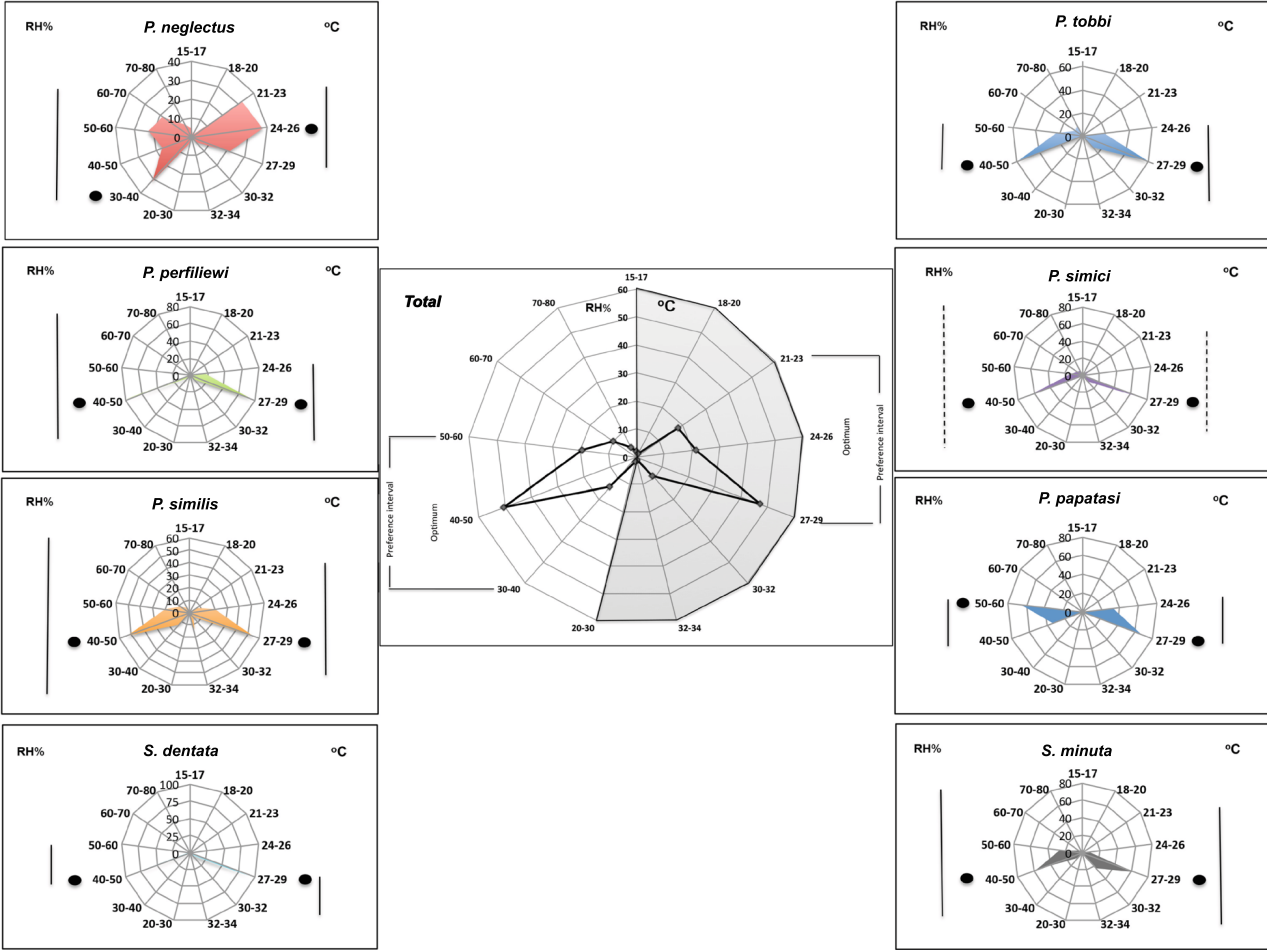
Species-specific relationships between the abundance of specimens and on-site recorded climatic variables. *Key*: black dot, optimum temperature and humidity ranges; vertical line: preference interval

Figure 3 shows the species-specific relationships between sand fly abundance and on-site recorded climatic variables, temperature (°C) and relative humidity (RH%), in the study area. In general, temperature and humidity preferences of aggregate sand fly populations was found between 21–29 °C. Nevertheless, there was a significant difference in terms of preference ranges of mentioned climatic factors between species (*P* > 0.001). While 27–29 °C and 40–50 RH% were determined the optimum for 7 species, a significant part of the *P. papatasi* population preferred 50–60 RH% as an optimum humidity range. Whilst *P. tobbi* was found to be a “narrowly distributed” species at the range of 24–30 °C (optimum of 27–29 °C ) and 40–60 RH% (optimum of 40–50 RH%), *Sergentomyia dentata* was the most “tightly distributed” sand fly species in terms of temperature and humidity ranges. The most important species on the islands, *P. neglectus*, was indisputably exposed as the most adapted “generalist” species in the study area with a very high reaction norm. Even lower temperature and humidity ranges were favorable to *P. neglectus*.

### Relationship between sand fly abundance in positive CDC traps and altitude, temperature and relative humidity ranges in the study area

Table 6 depicts the percentage of traps with sand flies and CDC sand fly abundance in positive traps according to altitude, relative humidity, temperature and island. The highest sand fly median (15 specimens) corresponded to altitudes between 300 and 400 masl, at temperatures of 21–23 °C (9 specimens) and 30–32 °C (10 specimens) and 41–50 RH%. However, the sand fly median was much lower (3 specimens) at 401–500 and 501–600 masl, at 60–70 RH% (2 specimens) and at 18–20 °C (3 specimens). Further analysis of the relationship between sand fly abundance in positive light traps, and on-site recorded temperature and relative humidity is also shown in Table 6. The percentage of positive light traps was strongly associated with increasing temperature. However, associations between RH% and light trap catches were less clear. While the percentage of positive traps seemed to be slightly associated with decreasing humidity, the relationship was not linear at lower humidity ranges of 41–50 RH%.

**Table 6:** Sand fly distribution according to altitude, relative humidity, temperature and island

Variable	Level	No. of traps	% positive traps	Sand fly distribution in positive traps (abundance)							
				No. of sand flies	Mean	Min.	25%	Median	75%	Max.	Interquartile range
Altitude (masl)	0–100	120	65	915	12	1	2	5	10	131	8
	101–200	54	52	909	12	1	2	10	20	236	18
	201–300	44	61	466	12	1	2	8	22	126	20
	301–400	25	76	474	11	2	6	15	28	150	22
	401–500	11	91	35	12	1	2	3	12	45	10
	501–600	8	50	158	13	1	1	3	43	45	42
	600 <	3	0	0	na	na	na	na	na	na	na
Relative humidity (%)	20–30	1	1	40	na	na	na	na	na	na	na
	31–40	54	43	405	14	1	2	4	8	164	6
	41–50	59	87	1587	12	1	4	10	20	236	16
	51–60	53	81	573	12	1	2	6	12	50	10
	61–70	49	49	215	12	1	1	2	13	38	12
	71–80	49	63	137	12	1	2	7	14	143	12
Temperature (°C)	15–17	9	57	44	2	1	2	5	7	7	5
	18–20	17	40	26	4	1	1	3	5	11	4
	21–23	36	57	493	11	1	2	9	18	143	16
	24–26	95	56	715	12	1	1	5	18	164	17
	27–29	62	62	1326	13	1	2	5	14	150	12
	30–32	22	95	328	14	1	3	10	42	236	39
	32–34	24	83	22	13	1	3	9	20	98	17
Island	Anafi	14	64	281	21	1	4	11	39	164	35
	Andros	20	60	86	12	1	2	6	10	86	8
	Folegandros	18	33	9	1	1	1	1	2	3	1
	Ikaria	26	58	206	13	1	3	5	22	45	19
	Karpathos	33	58	549	22	1	4	10	23	143	19
	Leros	18	100	199	14	1	3	7	8	50	5
	Milos	29	66	131	10	1	2	3	18	41	16
	Nisyros	28	100	1219	14	1	9	18	45	236	36
	Patmos	21	76	110	13	1	2	4	10	20	8
	Santorini	28	18	17	1	1	2	2	6	8	4
	Sifnos	30	70	150	10	1	1	4	10	29	9

*Abbreviation*: *na* non-applicable

Whilst minimum and maximum sample size in positive traps set in the islands were found between 1 and 236, sand fly abundance in positive traps and interquartile ranges (IQR) according to altitude, relative humidity, temperature and island were found to be variable. Overall, sand fly abundance in positive light traps at different quartiles and quartile ranges according to altitudinal, temperature and humidity ranges are shown in Table 6. The minimum number of sand flies in positive traps for all variables was 1. The highest 25% Q, 50% Q and 75% Q were 6, 15 and 28, respectively, at 301–400 masl altitudinal range; the maximum number of sand flies was 236 at 101–200 masl altitude. These numbers were 4, 10 and 20 at 41–50 RH% humidity range (max. 236) and 3, 10, and 42 at 30–32 °C temperature range (maximum 236), respectively. The relationship between the islands examined and sand fly abundance in positive traps was also variable. A total of 1219 sand fly specimens was collected in 28 traps: the percentage of positive traps was 100%, the highest 25% Q (9), 50% Q (18) and 75% Q (45) was calculated in Nisyros.

## Discussion

Overall, nine sand fly species were collected in the eleven islands studied. The most predominant of these, *P. neglectus* (42.75% of the total catch), is the important vector of *L. infantum* in the Eastern Mediterranean. *Phlebotomus tobbi*, an important vector of *L. donovani* in Cyprus [22, 23] and Turkey [24] accounted for 5.28% of the specimens and *P. similis*, the suspected vector of *L. tropica* in Crete, accounted for 3.65% [10, 25]. Another known vector of *L. infantum*, *P. perfiliewi* [1] was found in small numbers (1.32%). *Phlebotomus papatasi*, the vector of *L. major* in the Middle East which causes zoonotic cutaneous leishmaniasis using certain rodents as reservoir hosts [26], was also present, but in a small proportion (0.41%) in three out of eleven islands.

Hundreds of islands are located in the Aegean Sea between Greece and Turkey. The bigger ones are inhabited by a small indigenous human population but, during the summer months, visitors increase population numbers considerably. As a consequence of wars in Syria, Afghanistan, Pakistan, Iraq, refugees come to some of these islands. Most households keep animals for self-consumption: poultry, sheep, goats, and sometimes a small number of horses, donkeys and very seldom, cows. Sheep and goat farms are found in most islands, as well as vegetable gardens.

In relation to mainland Greece, where visceral leishmaniasis appears in 41 out of the 54 prefectures and canine leishmaniasis occurs in 43 out of 54 [9], *P. neglectus* was reported in 11 of the 19 prefectures where sand fly trapping had been carried out, *P. tobbi* in 12 out of 19 and *P. perfiliewi* in 14 out of 19 [9]. At the same time, cases of cutaneous leishmaniasis caused by *L. tropica*, are increasing in Greece and *P. similis*, which is believed to be the potential vector of *L. tropica* in the island of Crete where studies have been carried out [10, 25], was found in 11 of the 19 prefectures studied. These include five prefectures where cases have never been reported, implying the danger of introduction of this anthroponotic parasite to new regions. *Phlebotomus similis* is a sibling species of *P. sergenti* which is a proven vector of *L. tropica* causing cutaneous leishmaniasis in south and south-east Turkey [27, 28]. *Phlebotomus papatasi*, the vector of *L. major*, is abundant in most areas of Greece and Turkey (it was reported in 11 out of 19 prefectures in Greece), but no cases of zoonotic cutaneous leishmaniasis (ZCL) due to *L. major* are known in Greece [9]. They are, nevertheless, reported in Turkey [6]. This is possibly due to the fact that the known rodents playing the role of the reservoir host for this species are not present in Greece and no endemic rodents offer this potential to *L. major*. It is important that no such reservoir hosts are introduced in Greece from neighboring countries, such as Turkey, to avoid this parasite species becoming established.

In Turkey, visceral leishmaniasis caused by *L. infantum* and *L. donovani*, and cutaneous leishmaniasis caused by *L. tropica*, *L. infantum* and *L. major* were reported [27–30]; 22 species of sand flies are recorded, nine of which are proven or suspected vectors of *Leishmania* [30]. Of these, *P. neglectus*, *P. perfiliewi*, *P. papatasi*, *P. mascitti* and *P. alexandri* are common in both countries and, except *P. mascitti*, were also found in the Aegean islands.

Sand fly activity in the Aegean normally starts in April-May and ends in October [6] when the average temperature is optimal for sand flies (between 20–35 °C) and rain is scarce. Normally, the winds during May to October blowing over large parts of Greece, the Aegean Sea and Eastern Mediterranean are steady from northern to northwestern/northeastern directions and overall are dry, cool and seldom reach gale-force strength. Gale-force winds may occur as a result of thermal lows deepening over Turkey [31]. Such climatic conditions, the abundance of animals, domesticated, farm animals and wild life are favorable for sand flies.

From the data, it appears that *P. neglectus* and *S. minuta* preferred areas less than 1000 m distance from the coastline whilst *S. dentata* further than 2000 m. Only *P. simici* appeared at altitudes higher than 400 masl whereas *P. neglectus* and *S. dentata* were found at altitudes below 200 masl. Rural areas were more favorable for *P. neglectus*, urban for *P. similis* and *S. minuta*. *Phlebotomus tobbi* and *P. simici* preferred outdoor breeding and resting sites. The areas in which the different species were more recurrent could be explained by their trophic preferences (the presence of mammals and birds) and the environmental characteristics (Additional file 1: Table S1). An island ecosystem is a very particular one and difficult to compare to large inland landscapes. Small island size may allow sand flies to locate their meal sources in different microenvironments during evenings.

The distribution of the phlebotomine sand flies is highly disjunctive within their range, depending on local environmental factors such as precipitation and temperature, physical factors such as geographical barriers and habitat availability, and biotic factors such as the distribution and abundance of the vertebrate hosts [32, 33]. Although altitude is not a selective factor *per se*, biotic and abiotic properties of the environment are highly correlated with altitudinal gradients, the most obvious of which is climate [34]. In large parts of the southern Aegean islands, the climate is characteristic of much of the Mediterranean coast, with a mean temperature of 18–25 °C , relatively high humidity (40–70 RH%), and occasional summer showers that permit large populations of adult sand flies to develop. Moreover, the geographical and ecological richness of the islands provide numerous resting, and perhaps breeding sites for the flies. Seven and eight out of nine species collected from the study area were present in Nisyros and Milos islands, respectively, which are the closest islands to the mainlands, making them the most versatile of all the sites studied. This diversity may reflect the rocky nature and the warm, humid conditions of these islands combined with the influence of both the Mediterranean/Aegean elements and climate of the mainlands. The hilly areas represent interesting sites in terms of the discrete division observed in the distribution of sand fly species in domestic and rural habitats where most of the *Phlebotomus* species were restricted to domestic and rural habitats. This patchy distribution of adults is typical for most of the sand fly species because of their poor dispersal ability [35]. There was a remarkable difference in the diversity of the sand fly fauna, not only among islands but also altitudes. Like most ectotherms, the distribution of sand fly species is heavily dependent on temperature, therefore species situated along altitudinal gradients have to adapt to a variety of climatic conditions [36]. Therefore, temperature is one of the main factors preventing the spread of both visceral and cutaneous leishmaniasis [37]. This ecological factor varies with the altitude according to the thermal altitudinal gradient (-0.6 °C per 100 m). The possible relationship between leishmaniasis transmission and altitude may be closely related to many factors, such as the temperature suitable for the development of *Leishmania* in sand flies [38]. This study shows that altitude has an influence upon the spatial distribution of sand flies in the islands. Two associations of sand fly faunas were determined in the study area, the first one at lower altitudes between 0–400 masl and the second at relatively higher altitudes, between 400–600 masl. Species diversity was relatively high between 100–300 masl (highest at 200 masl) at slopes, corresponding to the transition between the coastline and the mountain. Alten et al. [6] found similar results related to altitudinal structuring of sand fly species in most of the Mediterranean countries, along with similar results by Prudhomme et al. [39] in southern France and Simsek et al. [40] in southern Turkey.

Four other associations were fixed among the islands in terms of the horizontal distribution of sand flies, the first one among Andros, Ikaria, Sifnos, the second among Nisyros and Milos, the third among Patmos and Leros, and the last among Folegandros, Karpathos and Anafi. In contrast to the lowest dominance indices, the species richness and diversity were found to be the highest in Nisyros and Milos which are the closest to the West (Milos) and to the East (Nisyros) of the mainlands. This corresponds to the horizontal transition between the East and West parts of the study area since they are more influenced by the mainlands of Greece and Turkey (Fig. 1, Table 5). Among the 11 islands, Nisyros showed a different composition not only in terms of highest species richness and/or diversity but also in the percentage of positive traps and sand fly abundance in positive traps. The highest number of sand fly specimens trapped (1219) with 100% trap performance (28 traps) was found in this island. There was a statistically significant difference (*P* < 0.05) between Nisyros and other islands according to the number of sand fly catches in positive traps. The maximum number was collected in Nisyros (236) at 132 m altitude, 43 RH% and 30 °C; moreover, the highest quartile ranges were calculated in this island (Table 6). As mentioned above, among the examined islands, Nisyros is the closest island to Turkey. Depaquit et al. [41] presented a hypothesis regarding the dispersion and speciation of the genus *Paraphlebotomus* from an ancestral area in the Middle East *via* southeastern and Mediterranean Turkey through the Greek islands of the Aegean Sea. According to the generalized tracks shown in these studies, it appears probable that the dispersion of the important species *P. sergenti* and *P. similis,* which are sibling species, began somewhere in the Middle East. From the Middle-Eastern center of dispersion, a northern migration route could have been followed by the common ancestor to *P. jacusieli* and *P. similis*. Another route circumventing the Paratethys Sea by the North, could have been followed by *P. similis* (or its ancestor), a route undoubtedly used by sand flies of other subgenera, in particular *Adlerius*. Indeed, taking into account the distribution of *P. similis* around the Black Sea and at the eastern edge of the Mediterranean Sea, an initial migration using the traditional routes of the Bosporus and of the South Aegean Islands seems probable. Depaquit et al. [20] showed that *P. sergenti* and *P. similis* are demonstrably monophyletic. Turkey is the only country in which both species are present; found in high plateaus of central Anatolia and in areas in the Aegean part of Anatolia close to Greek islands included in this study. Simsek et al. [40] and Aytekin et al. [42] made very important contributions to this hypothesis and to the distribution routes of sand fly species from eastern Anatolia to the western part of the Mediterranean Sea and to the Aegean Sea. Simsek et al. [40] reported high sand fly diversity, species richness, high microhabitat variability between Taurus and Anti-Taurus mountain series, displaying very suitable breeding habitats to sand fly species between East-West and North-South parts of Anatolia and also proved that neither altitude nor mountain series are physical barriers for sand fly dispersion on the East-West direction in Anatolia. Aytekin et al. [42] also confirmed uninterrupted dispersal of sand fly species on the East-West direction in Anatolia using geometric morphometric analyses, such as the wing shape and size of local populations of *P. sergenti*, *P. tobbi* and *P. similis*. Even though there are three major mountain ranges that may serve as geographical barriers for distribution of different animal and plant species, they found no statistically significant differences in wing morphology in all examined specimens of local populations collected from Aegean and Mediterranean coasts and eastern Anatolia because of determined gaps allowing dispersion between the barriers [42]. These results show that the barriers are not sufficient to stop gene flow among local populations of sand flies. Overall, these studies concluded that Anatolia has a very high species diversity (23 identified species) and that the species can uninterruptedly disperse in an East-to-West direction reaching the Aegean coast of Anatolia and the Greek islands located in the Aegean Sea due to the presence of transitional gaps between the regions of Mediterranean Anatolia. We assume that, since there is very high faunistic similarity between mainland Turkey and Nisyros (the island closest to Turkey in the study area) as well as high species richness, diversity and abundance rates of species, Nisyros is one of the main transition islands in the south Aegean Sea between the two mainlands and is the first stepping-stone for the dispersion of sand flies between Turkey and Greece. Kasap et al. [43, 44] showed this movement for *P. neglectus* as well as for species of the subgenus *Transphlebotomus*. According to analyses in these studies, members of *Transphlebotomus* and partly of *Larroussius* have been diversifying in the Aegean and Mediterranean regions for 10 million years, mainly driven by the old paleogeographic events that took place around these regions.

Canine leishmaniasis appears to be common in most Greek Aegean islands, with a seropositivity of 19.33% [9] but increasing, and human visceral leishmaniasis has being reported from some of the islands. However, since patients with serious health problems go to the country’s central hospitals for diagnosis and treatment, data on the number of patients infected on the islands is not available. Important vectors of *Leishmania* were found in all islands studied which represent a risk for parasite transmission to humans and dogs and the danger of maintaining new *Leishmania* spp. if introduced to the area. The increase in population numbers during the summer months and the arrival of refuges, in addition to the possibility of new sand fly and rodent species being introduced by wind, boats and aircraft, presents a complex situation which should be monitored to decrease the risk of *Leishmania* transmission and the spread of the leishmaniases in the area.

The abundance of a sand fly species is not by itself sufficient to incriminate it as a vector. Some populations of suspected vectors appear to be small, while others seem to have too short a season to maintain the circulation of a *Leishmania* [1]. The present findings show that the study area, with favorable topographical and climatic conditions for sand flies, harbors important vector species, such as *P. neglectus*, *P. tobbi*, *P. similis*, *P. perfiliewi* and *P. papatasi.* The high population abundance of *P. neglectus* represent a risk for *L. infantum* transmission in all islands. The presence of *P. tobbi* in 6 out of 11 islands constitutes a risk for the introduction of *L. donovani*, an anthroponotic species causing visceral leishmaniasis and cutaneous leishmaniasis in Cyprus [22] and Turkey [45]. The same danger stands for the anthroponotic *L. tropica* because its suspected vector, *P. similis*, was found in 10 out of the 11 islands studied and where cutaneous leishmaniasis cases have not been reported so far. This parasite species is found in the Ionian Islands, Crete and in areas on the coast of the Aegean Sea in Turkey. Already, refugees have arrived in Crete and Cyprus with skin lesions due to *L. tropica* [10, unpublished data]. The Greek Aegean islands are at high risk for the introduction of these anthroponotic diseases because the vectors are present and infected people may start the transmission cycle of the parasite.

## Conclusions

To the best of our knowledge, we have documented for the first time the presence of nine sand fly species and their distribution, diversity and abundance in 11 Greek islands of the Aegean Sea. The study indicated that these islands maintain a rich sand fly fauna which includes important vectors of *Leishmania* spp. representing a risk for parasite transmission to humans and dogs and the danger of maintaining new *Leishmania* spp. if introduced to the area. The distribution of the phlebotomine sand flies was highly disjunctive within their range, depending on local environmental factors such as precipitation and temperature, physical factors such as geographical barriers, habitat availability and biotic factors on the islands. There was a remarkable difference in the diversity of sand fly fauna, not only among islands but also in relation to altitude. We assume that, since there is very high faunal similarity between mainland Turkey and Nisyros (the island closest to Turkey in the study area) as well as high species richness, diversity and abundance of the species, Nisyros is one of the main transition islands in the South Aegean Sea between the two mainlands and is the first stepping-stone island for the dispersion of sand flies between Turkey and Greece.

## Additional file

Additional file 1: Table S1. Risk factors using the multivariate logistic regression model (using SPSS 22). (DOCX 21 kb)

## Abbreviations

CanLei: canine leishmaniasis
CDC: centre of disease control
CL: cutaneous leishmaniasis
*cox*1: cytochrome *c* oxidase subunit 1 gene
GPS: geographical positioning system
masl: meters above sea level
PCR: polymerase chain reaction
Q: quartile
VL: visceral leishmaniasis
ZCL: zoonotic cutaneous leishmaniasis

## References

[1] Killick-Kendrick R (1990). Phlebotomine vectors of the leishmaniases: a review. Med Vet Entomol..

[2] Chamberlin J, Laughlin LW, Romero S, Solórzano N, Gordon S, Andre RG (2002). Epidemiology of endemic *Bartonella bacilliformis*: a prospective cohort study in a Peruvian mountain valley community. J Infect Dis..

[3] Alwassouf S, Christodoulou V, Bichaud L, Ntais P, Mazeris A, Antoniou M (2016). Seroprevalence of sand fly-borne phleboviruses belonging to three serocomplexes (sand fly fever Naples, sand fly fever Sicilian and Salehabad) in dogs from Greece and Cyprus using neutralization test. PLoS Negl Trop Dis..

[4] Bichaud L, Izri A, de Lamballerie X, Moureau G, Charrel RN (2014). First detection of Toscana virus in Corsica, France. Clin Microbiol Infect.

[5] Bichaud L, Souris M, Mary C, Ninove L, Thirion L, Piarroux RP (2011). Epidemiologic relationship between Toscana virus infection and *Leishmania infantum* due to common exposure to *Phlebotomus perniciosus* sand fly vector. PLoS Negl Trop Dis..

[6] Alten B, Maia C, Afonso MO, Campino L, Jiménez M, González E (2016). Seasonal dynamics of phlebotomine sand fly species proven vectors of Mediterranean leishmaniasis caused by *Leishmania infantum*. PLoS Negl Trop Dis..

[7] Killick-Kendrick R, Rioux JA, Bailly M, Guy MW, Wilkes TJ, Guy FM (1984). Ecology of leishmaniasis in the south of France. 20. Dispersal of *Phlebotomus ariasi* Tonnoir, 1921 as a factor in the spread of visceral leishmaniasis in the Cevennes. Ann Parasitol Hum Comp..

[8] Lewis DJ (1971). Phlebotomid sandflies. Bull World Health Organ..

[9] Gunay F, Alten B, Ozsoy ED (2010). Estimating reaction norms for predictive population parameters, age specific mortality, and mean longevity in temperature-dependent cohorts of *Culex quinquefasciatus* Say (Diptera: Culicidae). J Vector Ecol..

[10] Ntais P, Sifaki-Pistola D, Christodoulou V, Messaritakis I, Pratlong F, Poupalos G (2013). Leishmaniases in Greece. Am J Trop Med Hyg..

[11] Christodoulou V, Antoniou M, Ntais P, Messaritakis I, Ivovic V, Dedet J-P (2012). Re-emergence of visceral and cutaneous leishmaniasis in the Greek island of Crete. Vector Borne Zoonotic Dis..

[12] Ivović V, Patakakis M, Tselentis Y, Chaniotis B (2007). Faunistic study of sandflies in Greece. Med Vet Entomol.

[13] Léger N, Pesson B, Madulo-Leblond G, Ferté H, Tselentis Y, Antoniou M (1993). Les phlebotomes de Crete. Resultats d’une enquete entomologique effectuee en Juillet 1988 et Aout 1989. Biol Gallo-hellenica..

[14] Léger N, Gramiccia M, Gradoni L, Madulo-Leblond G, Pesson B, Ferte H (1988). Isolation and typing of *Leishmania infantum* from *Phlebotomus neglectus* on the island of Corfu, Greece. Trans R Soc Trop Med Hyg..

[15] Xanthopoulou K, Anagnostou V, Ivovic V, Djurkovic-Djakovic O, Rogozi E, Sotiraki S (2011). Distribution of sandflies (Diptera, Psychodidae) in two Ionian islands and northern Greece. Vector Borne Zoonotic Dis..

[16] Lewis DJ (1982). A taxonomic review of the genus *Phlebotomus* (Diptera: Psychodidae). Bull Br Mus Nat Hist..

[17] Killick-Kendrick R, Tang Y, Killick-Kendrick M, Sang DK, Sirdar MK, Ke L (1991). The identification of female sandflies of the subgenus *Larroussius* by the morphology of the spermathecal ducts. Parassitologia..

[18] Folmer O, Black M, Hoeh W, Lutz R, Vrijenhoek R (1994). DNA primers for amplification of mitochondrial cytochrome *c* oxidase subunit I from diverse metazoan invertebrates. Mol Mar Biol Biotechnol..

[19] Hebert PDN, Ratnasingham S, de Waard JR (2003). Barcoding animal life: cytochrome *c* oxidase subunit 1 divergences among closely related species. Proc R Soc Lond B Biol Sci.

[20] Depaquit J, Ferté H, Léger N, Lefranc F, Alves-Pires C, Hanafi H, Maroli M (2002). ITS 2 sequences heterogeneity in *Phlebotomus sergenti* and *Phlebotomus similis* (Diptera, Psychodidae): possible consequences in their ability to transmit *Leishmania tropica*. Int J Parasitol..

[21] Hammer Ø, Harper DAT, Ryan PD (2006). PAST: Paleontological statistics software package for education and data analysis.

[22] Antoniou M, Haralambous C, Mazeris A, Pratlong F, Dedet J-P, Soteriadou K (2008). *Leishmania donovani* causing visceral or cutaneous leishmaniasis in Europe: Cyprus evidence is an alarm call. Lancet Infect Dis..

[23] Mazeris A, Soteriadou K, Dedet JP, Haralambous C, Tsatsaris A, Moschandreas J (2010). Leishmaniases and the Cyprus paradox. Am J Trop Med Hyg..

[24] Özbilgin A, Harman M, Karakuş M, Bart A, Töz S, Kurt Ö (2017). Leishmaniasis in Turkey: Visceral and cutaneous leishmaniasis caused by *Leishmania donovani* in Turkey. Acta Trop..

[25] Ntais P, Christodoulou V, Tsirigotakis N, Dokianakis E, Dedet J-P, Pratlong F (2014). Will the introduction of *Leishmania tropica* MON-58, in the island of Crete, lead to the settlement and spread of this rare zymodeme?. Acta Trop..

[26] Postigo JAR (2010). Leishmaniasis in the World Health Organization Eastern Mediterranean Region. Int J Antimicrob Agents..

[27] Ok Ü, Balcıoğlu İC, Özkan AT, Özensoy S, Özbel Y (2002). Leishmaniasis in Turkey. Acta Trop..

[28] Svobodová M, Alten B, Zídková L, Dvorák V, Hlavacková J, Myšková J (2009). Cutaneous leishmaniasis caused by *Leishmania infantum* transmitted by *Phlebotomus tobbi*. Int J Parasitol..

[29] Özbilgin A, Çulha G, Uzun S, Harman M, Topal SG, Okudan F (2016). Leishmaniasis in Turkey: first clinical isolation of *Leishmania major* from 18 autochthonous cases of cutaneous leishmaniasis in four geographical regions. Trop Med Int Health..

[30] Volf P, Ozbel Y, Akkafa F, Svobodova M, Votỳpka J, Chang K (2002). Sand flies (Diptera: Phlebotominae) in Sanliurfa, Turkey relationship of *Phlebotomus sergenti* with the epidemic of anthroponotic cutaneous leishmaniasis. J Med Entomol..

[31] Tyrlis E, Lelieveld J (2013). Climatology and dynamics of the summer Etesian winds over the eastern Mediterranean. J Atmospheric Sci..

[32] Cross ER, Newcomb WW, Tucker CJ (1996). Use of weather data and remote sensing to predict the geographic and seasonal distribution of *Phlebotomus papatasi* in southwest Asia. Am J Trop Med Hyg..

[33] Ghosh KN, Mukhopadhyay JM, Guzman H, Tesh RB, Munstermann LE (1999). Interspecific hybridization and genetic variability of *Phlebotomus* sandflies. Med Vet Entomol..

[34] Karan D, Dubey S, Moreteau B, Parkash R, David JR (2000). Geographical clines for quantitative traits in natural populations of a tropical drosophilid: *Zaprionus indianus*. Genetica..

[35] Munstermann LE, Morrison AC, Ferro C, Pardo R, Torres M (1998). Genetic structure of local populations of *Lutzomyia longipalpis* (Diptera: Psychodidae) in central Colombia. J Med Entomol..

[36] Telfer MG, Hassall M (1999). Ecotypic differentiation in the grasshopper *Chorthippus brunneus*: life history varies in relation to climate. Oecologia..

[37] Kuhn KG (1999). Global warming and leishmaniasis in Italy. Bull Trop Med Int Health..

[38] Rioux JA, Aboulker JP, Lanotte G, Killick-Kendrick R, Martini-Dumas A (1985). Écologie des leishmanioses dans le sud de la France-21 - Influence de la température sur le développement de *Leishmania infantum* Nicolle, 1908 chez *Phlebotomus ariasi Tonnoir*, 1921. Étude expérimentale. Ann Parasitol Hum Comp.

[39] Prudhomme J, Cassan C, Hide M, Toty C, Rahola N, Vergnes B (2016). Ecology and morphological variations in wings of *Phlebotomus ariasi* (Diptera: Psychodidae) in the region of Roquedur (Gard, France): a geometric morphometrics approach. Parasit Vectors..

[40] Simsek FM, Alten B, Caglar SS, Ozbel Y, Aytekin AM, Kaynas S (2007). Distribution and altitudinal structuring of phlebotomine sand flies (Diptera: Psychodidae) in southern Anatolia, Turkey: their relation to human cutaneous leishmaniasis. J Vector Ecol..

[41] Depaquit J, Ferte H, Leger N, Killick-Kendrick R, Rioux J-A, Killick-Kendrick M (2000). Molecular systematics of the phlebotomine sandflies of the subgenus *Paraphlebotomus* (Diptera, Psychodidae, *Phlebotomus*) based on ITS2 rDNA sequences. Hypotheses of dispersion and speciation. Insect Mol Biol..

[42] Aytekin AM, Alten B, Caglar SS, Ozbel Y, Kaynas S, Simsek FM (2007). Phenotypic variation among local populations of phlebotomine sand flies (Diptera: Psychodidae) in southern Turkey. J Vector Ecol..

[43] Kasap OE, Votýpka J, Alten B (2013). The distribution of the *Phlebotomus major* complex (Diptera: Psychodidae) in Turkey. Acta Trop..

[44] Kasap OE, Dvorak V, Depaquit J, Alten B, Votypka J, Volf P (2015). Phylogeography of the subgenus *Transphlebotomus* Artemiev with description of two new species, *Phlebotomus anatolicus* n. sp. and *Phlebotomus killicki* n. sp. Infect Genet Evol..

[45] Koltas IS, Eroglu F, Alabaz D, Uzun S (2014). The emergence of *Leishmania major* and *Leishmania donovani* in southern Turkey. Trans R Soc Trop Med Hyg..

