# Supplementary material for: Phlebotomine sand flies (Diptera: Psychodidae) in the Greek Aegean Islands: ecological approaches
Source: Parasit Vectors. 2018 Feb 20;11:97. doi: 10.1186/s13071-018-2680-4 (PMC5819154; doi:10.1186/s13071-018-2680-4)
Supplement: Supplementary file 1 — Risk factors using the multivariate logistic regression model (using SPSS 22). (DOCX 21 kb) [file 13071_2018_2680_MOESM1_ESM.docx]

**Additional file 1: Table S1.** Risk factors using the multivariate logistic regression model (using SPSS 22)

|  | ***P. neglectus*** | | ***P. tobbi*** | | ***P. simici*** | ***P. similis*** | | | ***S. minuta*** |  | | | ***S. dentata*** | |  |
| --- | --- | --- | --- | --- | --- | --- | --- | --- | --- | --- | --- | --- | --- | --- | --- |
| **Variable** | **Odds ratio (95 % CI)** | **p - value** | **Odds ratio (95 % CI)** | **p - value** | **Odds ratio (95 % CI)** | **p - value** | **Odds ratio (95 % CI)** | **p - value** | **Odds ratio**  **(95 % CI)** | | **p - value** | **Odds ratio (95 % CI)** | | **p - value** | |
| **Distance from sea** |  |  |  |  |  |  |  |  |  | |  |  | |  | |
| **0-1000 m** | 1 | <0.0001 |  | N.S. |  | N.S. |  | N.S. | 1 | | <0.0001 | 1 | | <0.0001 | |
| **1000-2000 m** | 1.17 (0.91 - 1.51) | 0.227 |  |  |  |  |  |  | 0.63 (0.46 - 0.85) | | 0.003 | 0.31 (0.11 - 0.89) | | 0.03 | |
| **≥2000 m** | 0.43 (0.32 - 0.58) | <0.0001 |  |  |  |  |  |  | 0.42 (0.31 - 0.59) | | <0.0001 | 16.89 (9.42 - 30.29) | | <0.0001 | |
| **Altitude** |  |  |  |  |  |  |  |  |  | |  |  | |  | |
| **0-200 m** | 1 | 0.008 |  | N.S. | 1 | <0.0001 |  | N.S. |  | | N.S. | 1 | | 0.012 | |
| **200-400 m** | 1.06 (0.83 - 1.36) | 0.647 |  |  | 2.40 (1.85 – 3.12) | <0.0001 |  |  |  | |  | 0.28 (0.12 - 0.65) | | 0.003 | |
| **≥400 m** | 0.35 (0.18 - 0.70) | 0.003 |  |  | 4.34 (2.35 – 8.01) | <0.0001 |  |  |  | |  | 0.84 (0.12 - 5.75) | | 0.860 | |
| **Vegetation** |  |  |  |  |  |  |  |  |  | |  |  | |  | |
| **Presence of trees** | 1 | <0.0001 | 1 | 0.005 | 1 | <0.0001 | 1 | <0.0001 | 1 | | <0.0001 |  | | N.S. | |
| **Absence of trees** | 0.18 (0.14 - 0.25) |  | 2.08 (1.25 – 3.48) |  | 2.44 (1.81 – 3.29) |  | 5.74 (3.18 - 10.37) |  | 1.87 (1.33 - 2.62) | |  |  | |  | |
| **Area** |  |  |  |  |  |  |  |  |  | |  |  | |  | |
| **Rural** | 1 | <0.0001 |  | N.S. |  | N.S. | 1 | <0.0001 | 1 | | <0.0001 |  | | N.S. | |
| **Sub-urban** | 0.15 (0.05 - 0.40) | <0.0001 |  |  |  |  | 37.84 (14.28 - 100.25) | <0.0001 | 1.02 (0.36 - 2.87) | | 0.969 |  | |  | |
| **Urban** | 0.07 (0.02 - 0.25) | <0.0001 |  |  |  |  | 51.88 (10.70 - 251.51) | <0.0001 | 9.67 (4.03 - 23.21) | | <0.0001 |  | |  | |
| **Domestic Animals** |  |  |  |  |  |  |  |  |  | |  |  | |  | |
| **Absence of animals** | 1 | <0.0001 | 1 | 0.002 | 1 | <0.0001 | 1 | <0.0001 | 1 | | <0.0001 | 1 | | <0.0001 | |
| **Poultry** | 8.68 (6.71 - 11.23) | <0.0001 | 0.51 (0.32 - 0.82) | 0.006 | 0.58 (0.44 - 0.76) | <0.0001 | 0.05 (0.02 - 0.13) | <0.0001 | 0.35 (0.25 - 0.49) | | <0.0001 | 0.09 (0.03 - 0.25) | | <0.0001 | |
| **Horses** | 2.21 (1.29 – 3.79) | 0.004 | 0.86 (0.30 - 2.45) | 0.780 | 0.63 (0.34 - 1.16) | 0.135 | 1.82 (0.66 - 5.02) | 0.247 | 1.48 (0.80 - 2.75) | | 0.210 | 0.25 (0.03 - 2.06) | | 0.199 | |
| **Other animals** | 10.46 (7.38 - 14.83) | <0.0001 | 0.28 (0.14 - 0.58) | 0.001 | 0.31 (0.20 - 0.48) | <0.0001 | 0.29 (0.14 - 0.65) | 0.002 | 0.53 (0.36 - 0.80) | | 0.002 | 0.11 (0.03 - 0.41) | | 0.001 | |
| **Indoor/outdoor** |  |  |  |  |  |  |  |  |  | |  |  | |  | |
| **indoor** | 1 | 0.046 | 1 | 0.022 | 1 | <0.0001 | 1 | <0.0001 | 1 | | <0.0001 | 1 | | 0.031 | |
| **Outdoor** | 1.45 (1.01 - 2.09) |  | 2.32 (1.13 - 4.78) |  | 3.93 (2.48 – 6.21) |  | 0.21 (0.1 - 0.44) |  | 0.53 (0.37 - 0.75) | |  | 0.35 (0.13 - 0.91) | |  | |
| **Humidity** |  |  |  |  |  |  |  |  |  | |  |  | |  | |
| **30-60%** | 1 | <0.0001 |  | N.S. | 1 | <0.0001 | 1 | 0.001 | 1 | | <0.0001 | 1 | | 0.001 | |
| **≥60%** | 5.00 (3.88 - 6.43) |  |  |  | 0.61 (0.46 - 0.82) |  | 0.24 (0.11 - 0.54) |  | 0.22 (0.15 - 0.33) | |  | 0.19 (0.07 - 0.52) | |  | |
| **Temperature** |  |  |  |  |  |  |  |  |  | |  |  | |  | |
| **<20ᵒC** | 1 | 0.007 | 1 | 0.001 | 1 | <0.0001 | 1 | 0.006 | 1 | | 0.003 |  | | N.S. | |
| **20-30ᵒC** | 0.35 (0.18 - 0.69) | 0.002 | 0.19 (0.07 – 0.49) | 0.001 | 1.37 (0.70 – 2.66) | 0.355 | 0.33 (0.06 - 1.88) | 0.212 | 14.65 (1.77 - 121.31) | | 0.013 |  | |  | |
| **≥30ᵒC** | 0.32 (0.16 - 0.66) | 0.002 | 0.28 (0.10 – 0.78) | 0.015 | 3.05 (1.52 – 6.13) | 0.002 | 0.12 (0.02 - 0.75) | 0.023 | 20.19 (2.43 - 167.67) | | 0.005 |  | |  | |
